# Loss of liver-specific and sexually dimorphic gene expression by aryl hydrocarbon receptor activation in C57BL/6 mice

**DOI:** 10.1371/journal.pone.0184842

**Published:** 2017-09-18

**Authors:** Rance Nault, Kelly A. Fader, Jack R. Harkema, Tim Zacharewski

**Affiliations:** 1 Department of Biochemistry & Molecular Biology, Michigan State University, East Lansing, MI, United States of America; 2 Institute for Integrative Toxicology, Michigan State University, East Lansing, MI, United States of America; 3 Pathobiology & Diagnostic Investigation, Michigan State University, East Lansing, MI, United States of America; INRA, FRANCE

## Abstract

The aryl hydrocarbon receptor (AhR) is a highly conserved transcription factor that mediates a broad spectrum of species-, strain-, sex-, age-, tissue-, and cell-specific responses elicited by structurally diverse ligands including 2,3,7,8-tetrachlorodibenzo-*p*-dioxin (TCDD). Dose-dependent effects on liver-specific and sexually dimorphic gene expression were examined in male and female mice gavaged with TCDD every 4 days for 28 or 92 days. RNA-seq data revealed the coordinated repression of 181 genes predominately expressed in the liver including albumin (3.7-fold), α-fibrinogen (14.5-fold), and β-fibrinogen (17.4-fold) in males with corresponding AhR enrichment at 2 hr. Liver-specific genes exhibiting sexually dimorphic expression also demonstrated diminished divergence between sexes. For example, male-biased *Gstp1* was repressed 3.0-fold in males and induced 4.5-fold in females, which were confirmed at the protein level. Disrupted regulation is consistent with impaired GHR-JAK2-STAT5 signaling and inhibition of female specific CUX2-mediated transcription as well as the repression of other key transcriptional regulators including *Ghr*, *Stat5b*, *Bcl6*, *Hnf4a*, *Hnf6*, *Foxa1/2/3*, *and Zhx2*. Attenuated liver-specific and sexually dimorphic gene expression was concurrent with the induction of fetal genes such as alpha-fetoprotein. The results suggest AhR activation causes the loss of liver-specific and sexually dimorphic gene expression producing a functionally “de-differentiated” hepatic phenotype.

## Introduction

2,3,7,8-Tetrachlorodibenzo-*p*-dioxin (TCDD) is a persistent environmental contaminant that bioaccumulates and elicits a broad spectrum of biochemical and toxic effects. These effects are largely mediated by the aryl hydrocarbon receptor (AhR), a ligand-activated transcription factor [1]. Following ligand binding, cytosolic AhR dissociates from its chaperone proteins (e.g., HSP90, XAP2, and P23) and translocates to the nucleus where it dimerizes with the aryl hydrocarbon receptor nuclear translocator (ARNT). This complex interacts with dioxin response elements (5’-GCGTG-3’) to elicit differential gene expression. AhR-mediated gene expression changes also involve interactions with non-consensus sequences or tethering to other transcription factors [2, 3].

The liver is a primary target of TCDD-elicited pathologies including the induction of fatty liver disease [4, 5], impaired cellular proliferation [6], altered iron and bile acid homeostasis [7], reprogramming of carbohydrate and amino acid metabolism [8], coagulation cascade activation [9], and development of hepatocellular carcinoma (HCC) [10]. Moreover, AhR null mice show impaired liver development [11]. Although many phenotypic and underlying hepatic transcriptomic responses of AhR activation are conserved across sexes, differences in sensitivities between male and female mice are reported [12, 13]. These differences warrant further investigation as the role of sex in disease development and toxicity has not been adequately investigated, particularly in liver, one of the most sexually dimorphic organs [14].

Sexually dimorphic gene expression is closely associated with liver-specific gene regulation. It is largely mediated by the growth hormone (GH) signaling cascade (GHR-JAK2-STAT5) in mice, rats, and humans [15–18]. In males, the pulsatile secretion of GH by the pituitary is under gonadal steroid control while females exhibit more stable GH levels due to continuous secretion [19]. GH binding to the GH receptor (GHR), JAK2 activation, and STA5b phosphorylation results in intermittent or persistent STA5b activation and DNA binding [19]. The female-specific transcription factor, CUX2, and the expression of other hepatocyte nuclear factors (e.g., Hepatocyte Nuclear Factor 4 α;HNF4α, B-Cell CLL/Lymphoma 6; BCL6, Zinc Fingers and Homeoboxes 2; ZHX2) also influence GHR-JAK2-STAT5 signaling and serves an important role in determining sexually dimorphic xenobiotic metabolism gene expression [19, 20]. These same transcription factors are also implicated in liver development and hepatocyte differentiation [21].

AhR agonists impair GHR-JAK2-STAT5 signaling in mice and rats [22–25] and are reported to cause feminization of male mouse liver gene expression [26]. To further investigate AhR-mediated hepatotoxicity, the effects of TCDD on liver-specific and sexually dimorphic gene expression were examined using published hepatic RNA-seq datasets from male and female mice orally gavaged with 0–30 μg/kg TCDD every 4 days for 28 or 92 days (females only) [7, 27, 28]. Overall, TCDD repressed sexually-dimorphic gene expression in male and female livers yielding a more ambiguous phenotype (e.g. male and female hepatic gene expression converged to sexually ambiguous profiles). In addition, TCDD elicited the loss of liver-specific gene expression while inducing gene expression associated with liver progenitor cells producing a functionally “de-differentiated” hepatocyte phenotype. These changes are consistent with the disruption of GHR-JAK2-STAT5, CUX2, and HNF signaling. The cumulative loss of the associated functions may compromise liver function and contribute to TCDD-elicited hepatotoxicity.

## Materials and methods

### Animals

The animals, study design, and analysis of transcriptomic and ChIP-seq data have been previously described [29]. Briefly, postnatal day 25 (PND25) C57BL/6 mice were obtained from Charles River Laboratories (Portage, MI). Females were housed in polycarbonate cages with cellulose fiber chips (Aspen Chip Laboratory Bedding, Warrensberg, NY) and males in Innovive cages (Innovive inc., San Diego, CA) with ALPHA-dri (Shepherd Specialty Papers, Chicago, IL) bedding at 30–40% humidity, 23°C, and a 12h light/dark cycle were fed *ad libitum* (Harlan Teklad 22/5 Rodent Diet 8940, Madison, WI) with free access to water. Housing conditions between sexes differ due to changes within the animal care facilities at Michigan State University. No significant differences in TCDD-elicited responses due to these changes have been observed. On PND 28 and every following 4th day (d), animals were orally gavaged with 0.1 mL sesame oil or 0.01, 0.03, 0.1, 0.3, 1, 3, 10 and 30 μg/kg TCDD (Dow Chemical Company, Midland, MI) for a total of 28 or 92d (females only). Male mice exhibited greater sensitivity to TCDD and therefore were not treated for 92 days to avoid overt toxicity and death. Four days following the final dose mice were sacrificed by cervical dislocation. Liver samples were collected and immediately frozen in liquid nitrogen. Doses used compensate for the short study duration compared to lifelong cumulative human exposure from diverse AhR ligands, their bioaccumulative nature, and differences in metabolism and half-life. Furthermore, doses result in hepatic tissue levels that span human background serum concentrations reported in the United States, Germany, Spain, and the United Kingdom as well as serum levels in Viktor Yushchenko 4–39 months following intentional poisoning [30]. All procedures were approved by the All-University Committee on Animal Use and Care.

### Transcriptomic, AhR ChIP-seq, and putative dioxin response element data

Transcriptomic, AhR ChIP-seq, and computationally identified putative dioxin response element (pDRE) data were previously published [7, 8, 28]. Genes were considered differentially expressed when |fold-change| ≥ 1.5 and posterior probability values (P1(*t*)) ≥ 0.8 determined using an empirical Bayes approach [28]. Significant AhR ChIP-seq binding used a FDR ≤ 0.05. pDREs were considered functional when the matrix similarity score (MSS) ≥ 0.85. Sequencing data is available in the Gene Expression Omnibus (GEO; GSE62902, GSE81990, GSE87519, GSE97634, GSE97636). Sexually dimorphic genes were identified using RNA-seq datasets from male and female mice from vehicle treatment groups from the studies described above (GSE62902 and GSE87519) to account for age and other experimental factors. Following read mapping to the mouse reference genome (GRCm38) and gene count determinations [28], counts were transformed through variance stabilizing transformation (VST) using DESeq in R [31]. Data was normalized using a semi-parametric approach in SAS v9.3. An empirical Bayes approach was used to calculate posterior probabilities (P1(*t*)) values. Genes were considered sexually dimorphic if there was a |fold-change| ≥ 2.0 and P1(*t*) ≥ 0.8 difference between male and female transcriptomic data sets (S1 Table). Identification of over-represented transcription factor binding sites for ChIP-seq peaks was performed using Pscan-ChIP [32].

### Gene set enrichment and neighborhood gene expression regulation analyses

Gene set enrichment analysis (GSEA) [33] of sexually dimorphic and liver-specific genes was performed using a pre-ranked gene list based on the magnitude of the fold-change (largest induction to most repressed). The liver-specific gene set was identified using published microarray data representing 96 different tissues and cell types including adult male liver [34]. Tissue specificity was determined based on the difference of the gene in the tissue of interest and subtracting the highest signal in all other tissues (S1 Fig). Genes were considered liver-specific when the difference in microarray signal was ≥ 5,000 (S2 Table).

Genomic neighborhoods consisting of several nearby genes exhibiting similar differential expression, indicating putative shared regulation, was determined by calculating a running sum of log_2_ fold-changes. Firstly, the differentially expressed gene (|fold-change| ≥ 1.5) with smallest transcription start site (TSS) genomic coordinate was identified and that fold-change (in log_2_ space) was set as the initial score. The next differentially expressed gene based on TSS genomic coordinate was then found and that value (in log_2_ space) was added to the score. When a gene TSS was identified but did not exhibit a change in expression, the existing score was recorded. This process was continued across all genes in the chromosome, then all chromosomes. Scores were plotted based on the TSS coordinate and assessed for neighborhoods identified as dramatic increases (induction) or decreases (repression) revealing genome regions of closely located genes that may be co-regulated.

### Histopathology

Paraffin embedded livers were sectioned at 4 μm, placed on slides coated with 2% 3-Aminopropyltriethoxysilane, and dried at 56°C overnight. Slides were deparaffinized in Xylene and hydrated through descending grades of ethyl alcohol to distilled water then placed in Tris Buffered Saline pH 7.4 (Scytek Labs, Logan, UT) for 5 minutes (min). Endogenous peroxidase was blocked using 3% Hydrogen Peroxide/Methanol bath for 30 min followed by running tap and distilled water rinses. Standard micro-polymer complex staining steps were performed at room temperature on the IntelliPath™ Flex Autostainer. All staining steps are followed by TBS Autowash buffer (Biocare Medical, Concord, CA) rinses. After blocking for non-specific protein with Background Punisher (Biocare) for 10 min, sections were incubated with Polyclonal Rabbit anti—AFP (Protein Tech, 14550-1-AP; 1:100) in normal antibody diluent (NAD-Scytek) for 60 min. Following 30 min in ProMark Rabbit anti–Rodent™ Micro-Polymer (Biocare), reaction development was performed with Romulin AEC™ (Biocare) for 5 minutes and counterstained with CAT Hematoxylin (Biocare). Histological processing and staining was performed at the Michigan State University Investigative Histopathology Lab (https://humanpathology.natsci.msu.edu/).

### Protein determinations

Protein level were determined using a Wes capillary electrophoresis system (ProteinSimple, San Jose, CA) as previously described [8]. Liver samples were homogenized in radioimmunoprecipitation (RIPA) buffer supplemented with protease inhibitor cocktail. Total protein was quantitated by bicinchoninic acid assay. Equal amounts of protein were analyzed using antibodies for albumin (ALB; Abclonal A0353; 1:50), glutamate dehydrogenase 1 (GLUD1; Abclonal A7631; 1:100), glutathione S-transferase Pi 1 (GSTP1; Abclonal A5691; 1:50), hydroxyacid oxidase 1 (HAO1; Abclonal A6470; 1:50) and signal transducer and activator of transcription 5 (STAT5; Cell Signaling 9363; 1:50). Unlike traditional western blots, the Wes system quantitatively measures the chemiluminescence signal which were used to generate presented figures. Traces are provided as S2 Fig. Statistical analysis of protein levels in vehicle and treated mice were performed using the nonparametric Mann-Whitney’s U-test and considered significantly different when *P* ≤ 0.05.

## Results

### TCDD-mediated repression of liver-specific gene expression

**Fig 1:**
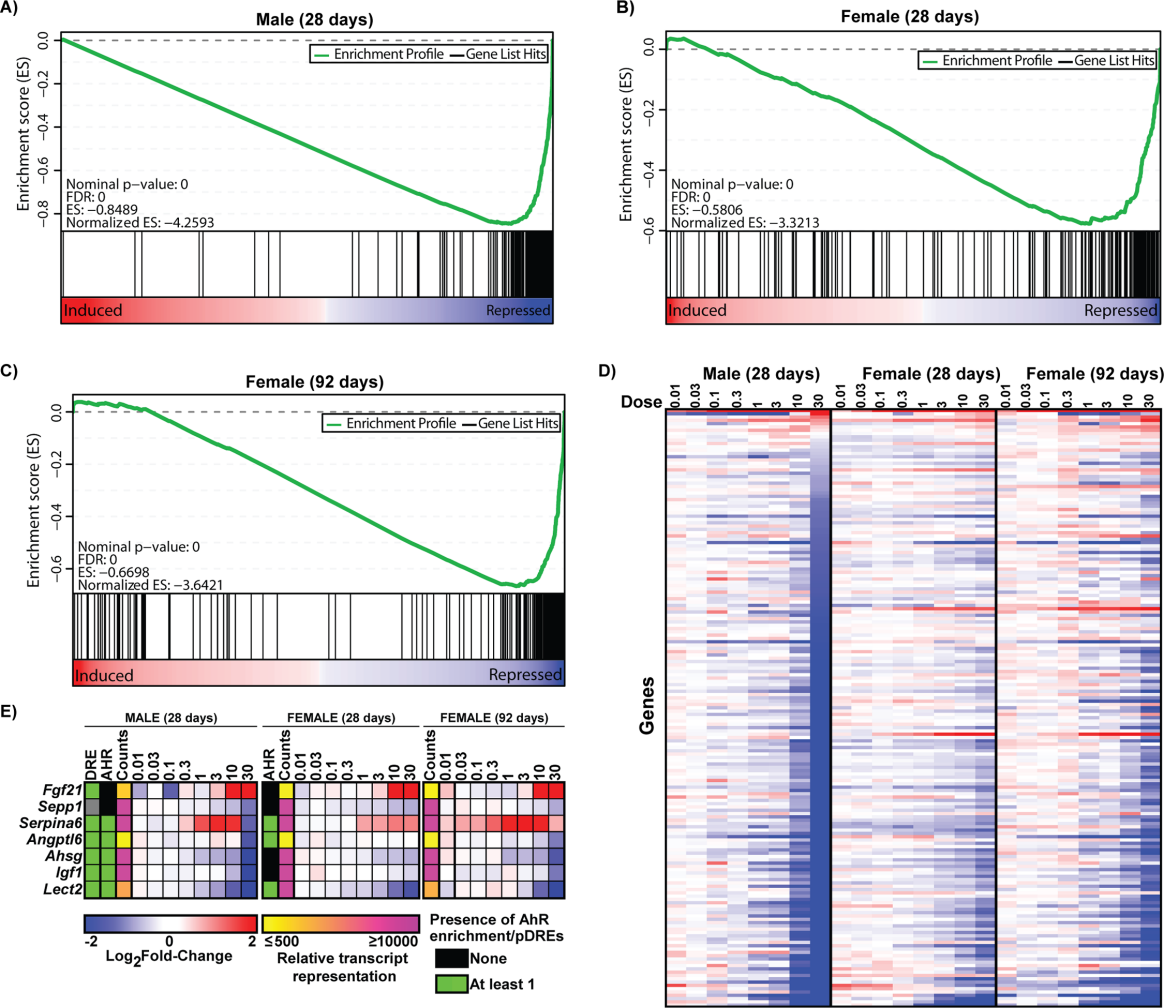
Gene expression changes of liver related genes. Gene set enrichment analysis (GSEA) of 181 liver specific-genes in (A) male and (B) female mice gavaged with 30 μg/kg TCDD every 4 days for 28 days, or (C) female mice gavaged with 30 μg/kg TCDD every 4 days for 92 days. The 181 liver-specific genes were identified using published microarray datasets representing 96 tissues/cell types [34]. Identification of liver-specific genes is described in materials and methods. TCDD elicited gene expression changes were ranked from most induced (left—red) to most repressed (right). Vertical black line represents identified liver-specific genes. The top panel (green line) represents a running-sum statistic (enrichment score) based on the lower panel, increasing when a gene is a member of the liver-specific gene set and decreasing when it is not. Enrichment scores increased most dramatically on the right indicating most of the liver-specific genes were repressed by TCDD. (D) Heat map of liver-specific gene expression changes elicited by TCDD. (E) Heatmap of TCDD-elicited repression of hepatokines. For heatmaps (D and E) blue indicates repression while red represents induction. The presence of pDREs (MSS ≥ 0.856) and hepatic AhR enrichment peaks (FDR ≤ 0.05) at 2h are shown as green boxes. Read count represents the maximum raw number of aligned reads to each transcript where yellow represents a lower level of expression (≤ 500 reads) and pink represents a higher level of expression (≥ 10,000).

**Fig 2:**
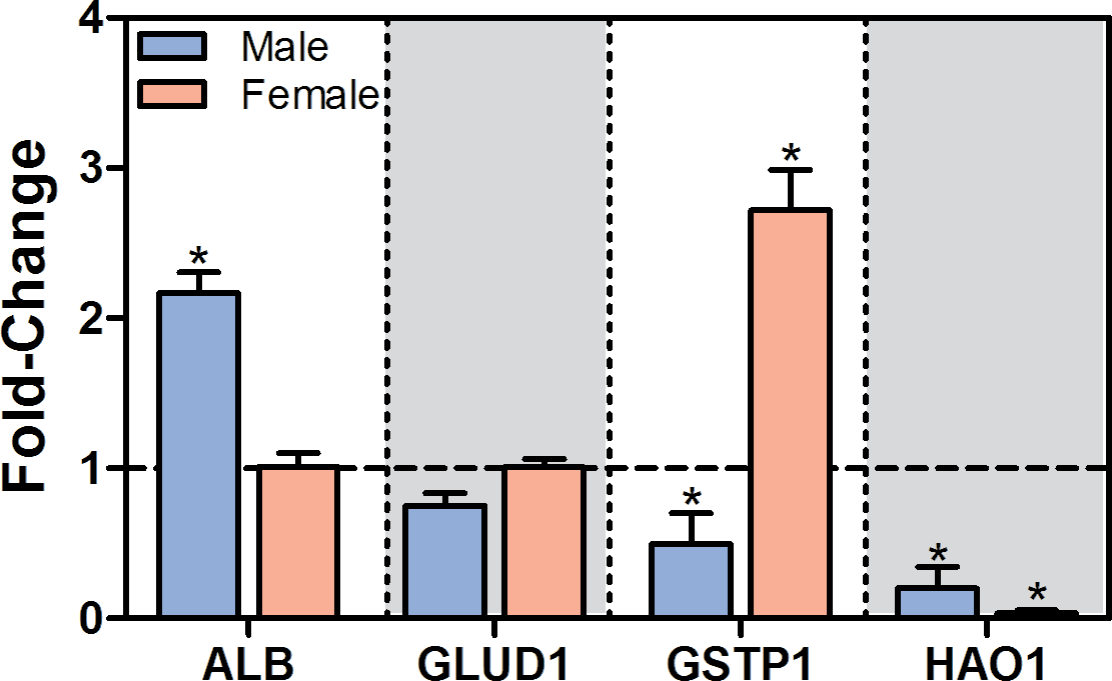
Hepatic protein levels changes by TCDD. Hepatic protein levels in male and female mice gavaged with sesame oil vehicle or 30 μg/kg TCDD every 4 days for 28 days were measured using the ProteinSimple Wes system. Bars represent mean log_2_fold-change + SEM for at least 4 animals (N = 4–5). Asterisks (*) indicate a significant difference (*P* ≤ 0.05) compared to vehicle control determined by Mann-Whitney U-test.

To further explore the effect of TCDD on hepatic gene expression, 181 genes primarily expressed in the liver were identified using 96 GeneAtlas MOE430 datasets representing tissue/cell types from naïve male mice [34]. These 181 genes are subsequently referred to as “liver-specific” based on a microarray signal difference of ≥5,000 units when comparing constitutive expression in liver to other non-hepatic tissues/cell types. Signal difference was used instead of fold change to capture only highly expressed genes. Our liver-specific gene set included previously identified liver-specific genes including albumin (*Alb*), methionine adenosyltransferase (*Mat1a*), fatty acid binding protein 1 (*Fabp1*), and fetuin-A (*Ahsg*). GSEA revealed repression by TCDD in both male and female mice at 28 and 92d suggesting an overall loss of liver-specific expression in both sexes (Fig 1A–1C). Repressed genes included *Alb*, *Mat1a*, glutamate dehydrogenase 1 (*Glud1*), and hydroxyacid oxidase 1 (*Hao1*) in both sexes. Hepatic HAO1 protein levels were also repressed in males while hepatic ALB levels increased contrary to its transcriptional repression (Fig 2). Liver-specific genes demonstrating increased expression in males and females included *Cyp1a2* and *Ugt2b35*, members of the AhR gene battery [35].

At 30 μg/kg, repressed genes included hepatokines, and secreted liver proteins with autocrine or paracrine activity, (e.g., *Lect2*–12.8-fold; *Igf1*–8.0-fold; *Ahsg* -4.9-fold, *Angptl6*–2.9-fold; *Sepp1*–2.1-fold) in male mice (Fig 1E). *Ahsg* and *Sepp1* were also among the 181 liver-specific genes and *Lect2*, *Ahsg*, and *Sepp1* were repressed 3.1- (92d), 1.5- (92d), and 1.6-fold (28 and 92d), respectively, in females. Interestingly, changes in hepatokine expression have been associated with obesity, type II diabetes, and nonalcoholic fatty liver disease (NAFLD) in rodents and humans consistent with the development and progression of NAFLD by TCDD in mice [1, 4, 5, 36]. Conversely, *Fgf21* which improves insulin resistance and reduces lipid accumulation [36] was induced 22.8- and 17.2-fold in male and female mice at 28d, respectively, while *Serpina6* (corticosteroid binding globulin; CBG) exhibited an inverted “U”-shaped dose-response in both sexes with 3.4- and 2.1-fold induction in male and female mice, respectively. *Fgf21* induction is a characterized AhR-mediated response [37]. *Serpina6* shows strong AhR ChIP-seq enrichment upstream of the TSS in male and female mice while *Angptl6* also had conserved AhR enrichment in both sexes (Fig 1E).

### Induction of liver progenitor cell marker alpha-fetoprotein

**Fig 3:**
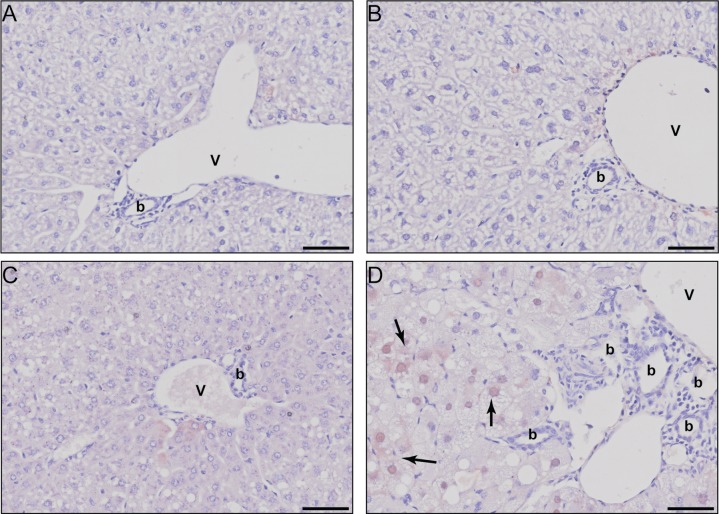
Immunohistochemical evaluation of hepatic AFP. Representative photomicrographs for AFP stained liver sections of (A) sesame oil vehicle treated females, (B) sesame oil vehicle treated males, (C) 30 μg/kg TCDD treated females, and (D) 30 μg/kg TCDD treated males. Scale bar represents 50 μm. The portal vein is designated by the letter V, bile ducts with the letter b, and AFP positive stained regions by solid black arrows.

**Fig 4:**
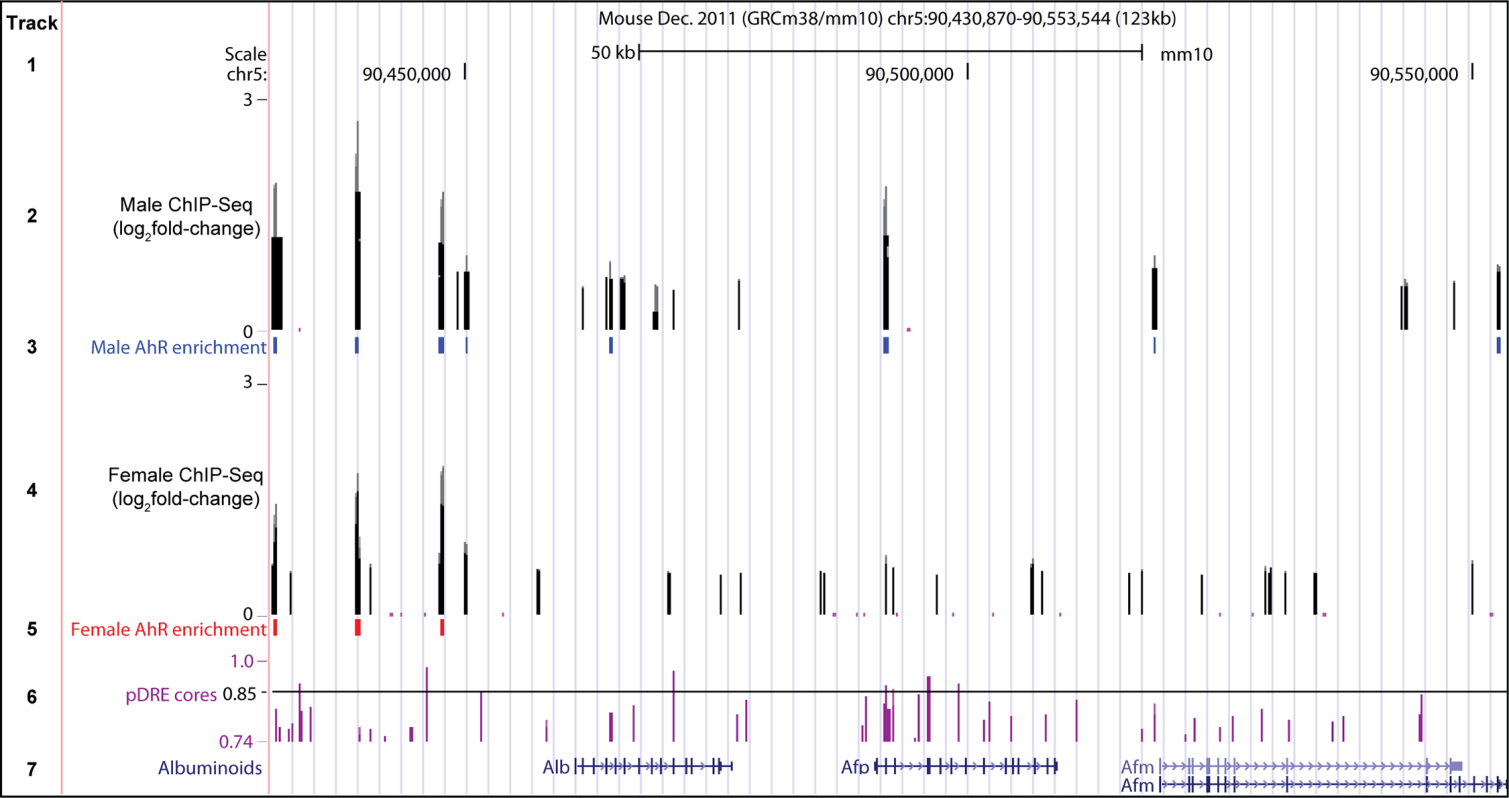
Albuminoid genomic region. Albuminoid genomic region including *Alb*, *Afp*, and *Afm*. UCSC genome browser tracks show (1) the scale, (2) male AhR ChIP-seq peaks at 2 h, (3) male AhR enriched sites (FDR ≤ 0.05), (4) female AhR ChIP-seq peaks, (5) female AhR enriched sites (FDR ≤ 0.05), (6) location of pDREs (diagonal line indicates pDREs with a matrix similarity score ≥0.85), and (7) location of *Alb*, *Afp* and *Afm* genes within the albuminoid genomic region. *Gc*, the fourth albuminoid, is located 1 Mb upstream of *Alb* (not shown). Tracks are available for visualization at http://dbzach.fst.msu.edu/index.php/publications/supplementary-data/.

The 1.6- and 15.0-fold induction of alpha-fetoprotein (*Afp;* 30 μg/kg) as well as 7.4- and 7.8-fold induction of TNF receptor superfamily 19 (*Tnfrsf19*; 30 μg/kg) in female and male mice, respectively, contrasts the overall repression of adult liver-specific gene expression. AFP is typically expressed during development in hepatic progenitor cells or hepatocellular carcinoma cells. Despite the lack of histological evidence of tumorigenesis, liver sections were evaluated for AFP expression. Increased cytosolic and intranuclear AFP staining was observed in male liver sections at 30 μg/kg TCDD including within hypertrophic differentiated hepatocytes showing lipid vacuolization, but not in female sections (Fig 3). The apparent difference in scale of histological images reflect the increased sensitivity of male mice to TCDD (Fig 3D). Expression of other albuminoids which play important roles in systemic transport such as albumin (*Alb*), afamin (*Afm*), and vitamin-D binding protein (*Gc*) were repressed 3.7-, 7.8-, and 4.3-fold, respectively, in male mice at 30 μg/kg. Repression of *Alb*, *Afm* and *Gc* was also observed in females following 92d of exposure, albeit to a lesser extent. Notably, *Alb*, *Afp*, *Afm* and *Gc* are sequentially co-localized on the same chromosome and arranged in the same transcriptional orientation in all mammals [38]. AhR enrichment was observed at 3 sites without pDREs ~ 13 – 30kb upstream of *Alb* in both male and female mice 2 h after TCDD treatment suggesting putative AhR-dependent co-regulation (Fig 4). The transcriptional repressor, zinc-finger and homeoboxes 2 (*Zhx2*), is also known to inhibit the fetal hepatic expression of *Afp*, *H19*, and *Gpc3* [20]. Accordingly, 30 μg/kg TCDD repressed *Zhx2* 1.6-fold while fetal markers *Afp*, *H19* and *Gpc3* were induced 15.0-, 2.9- and 2.5-fold, respectively.

### AhR enrichment at liver-specific genes

**Fig 5:**
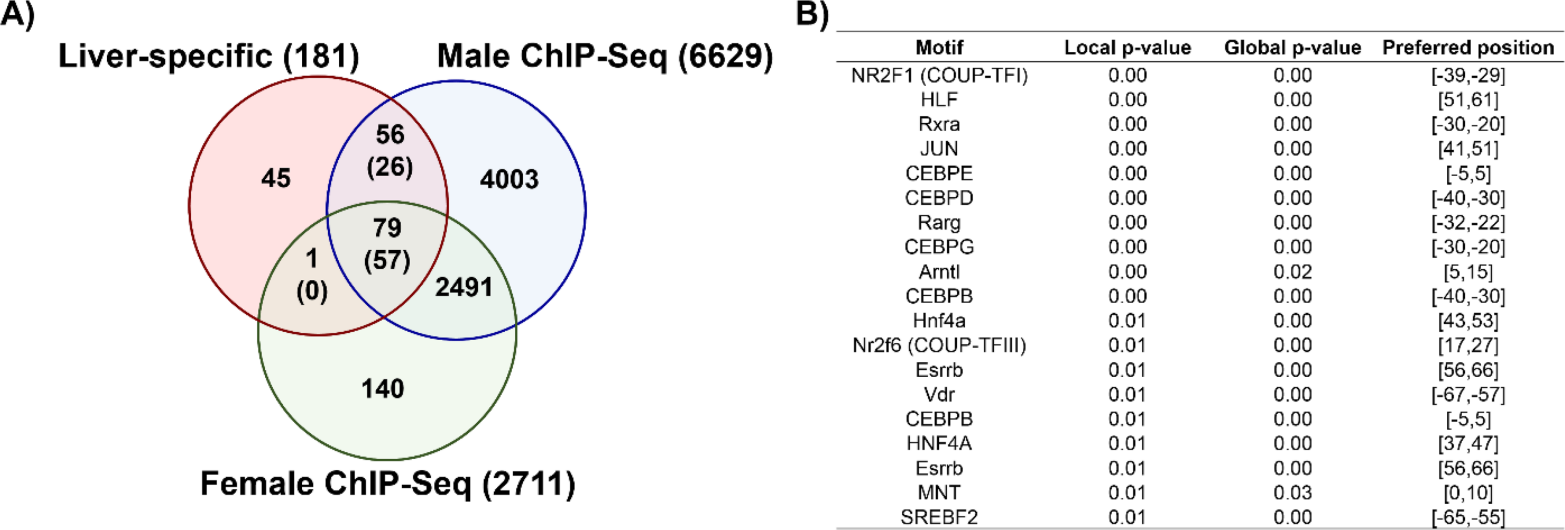
AhR enrichment and motif analyses. Comparison of AhR enrichment at liver-specific genes in male and female mice. (A) Values represent the number of unique genes in each segment of the Venn diagrams while numbers in parentheses show the number of AhR enriched regions containing a pDRE (matrix similarity score ≥ 0.85) for liver-specific genes. (B) Sequences beneath AhR enriched regions were queried for over-represented transcription factor binding motifs using Pscan-ChIP (Zambelli et al. 2013).

AhR enrichment was observed within 136 of the 181 liver-specific genes (79 in both sexes, 56 in males only, and 1 in females only; Fig 5A). pDREs were present in the majority of these binding sites (26 of 56 male sites, 57 of 79 male and female sites, and 0 of 1 female only sites), suggesting direct AhR-DRE-dependent regulation. Over-represented transcription factor motif analysis within AhR enriched regions identified binding sites for HNF4α, COUP-TFI and III, retinoic acid receptor (RXRA, RARG), CCAAT/Enhancer binding proteins (CEBPB, D, E, and G), and vitamin D receptor (VDR) (Fig 5B). In male mice, *Cebpd*, *Rarg*, and *Vdr* were induced 2.0-, 2.8-, and 2.1-fold, respectively, while *Cebpe* was repressed 4.1-fold at 30 μg/kg TCDD. Only *Rarg* was differentially expressed in females showing a 1.6-fold induction at 30 μg/kg TCDD. Also implicated in liver-specific gene expression were HNF6 (*Onecut1*) and HNF3 (*Foxa1*, *Foxa2*, *and Foxa3*) (repressed 5.6-, 1.7-, 1.6-, and 2.3-fold, respectively in males at 30 μg/kg) [39]. HNF6 and HNF3 repression is consistent with the 8.1-fold repression of transthyretin (*Ttr*), a male target gene. Smaller changes were observed in females after 92d but not at 28d. These results are consistent with proposed AhR-COUP-TFII-mediated repression [2].

### TCDD results in loss of sexually dimorphic gene expression

**Fig 6:**
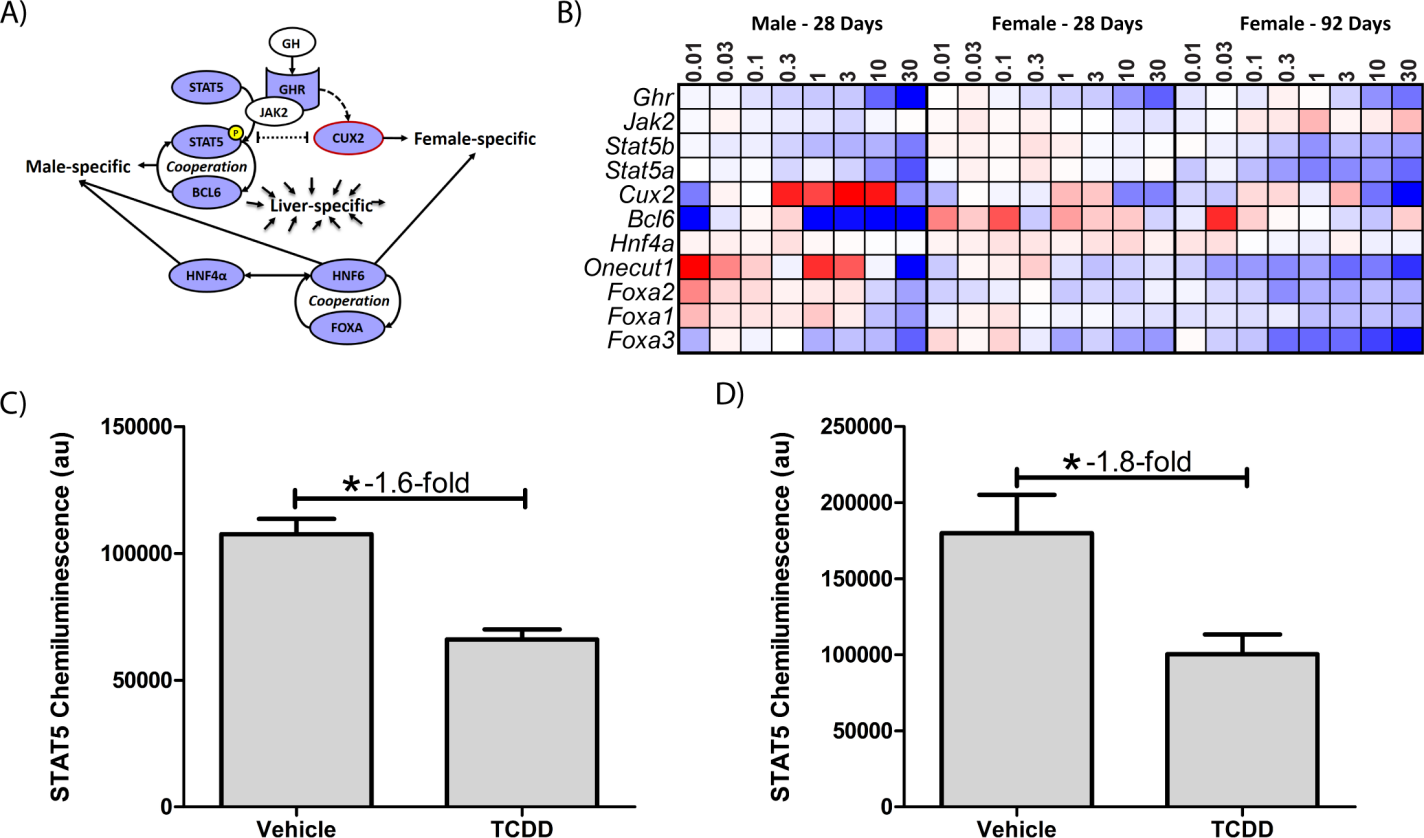
AhR-mediated changes in sexually dimorphic regulator expression. (A) GHR-JAK2-STAT5 signaling cascade interactions with liver-specific gene expression regulators [16, 18, 19, 43]. GH-mediated activation of GHR induces STAT5 phosphorylation promoting male-specific gene expression, and induction of female-specific CUX2 (red outline) which regulates female-specific gene expression. CUX2 and STAT5 compete for binding sites resulting in sexually dimorphic gene expression. BCL6, HNF4α, HNF6, and FOXA also regulate sexually dimorphic and liver-specific gene regulation. Blue identifies repressed genes while white represents genes unaffected by TCDD. (B) Heatmap of sexually dimorphic and liver-specific gene expression regulators (A) in males (28d) and females (28 and 92d). Genes in blue were repressed while red indicates induction. STAT5 protein levels were determined in (C) female and (D) male mice gavaged with 30 μg/kg TCDD every 4 days for 28 days. Bars represent mean ± SEM for at least 3 animals (N = 3–4). Asterisks (*) indicate a significant difference compared to vehicle control as determined by Mann-Whitney U-test.

**Fig 7:**
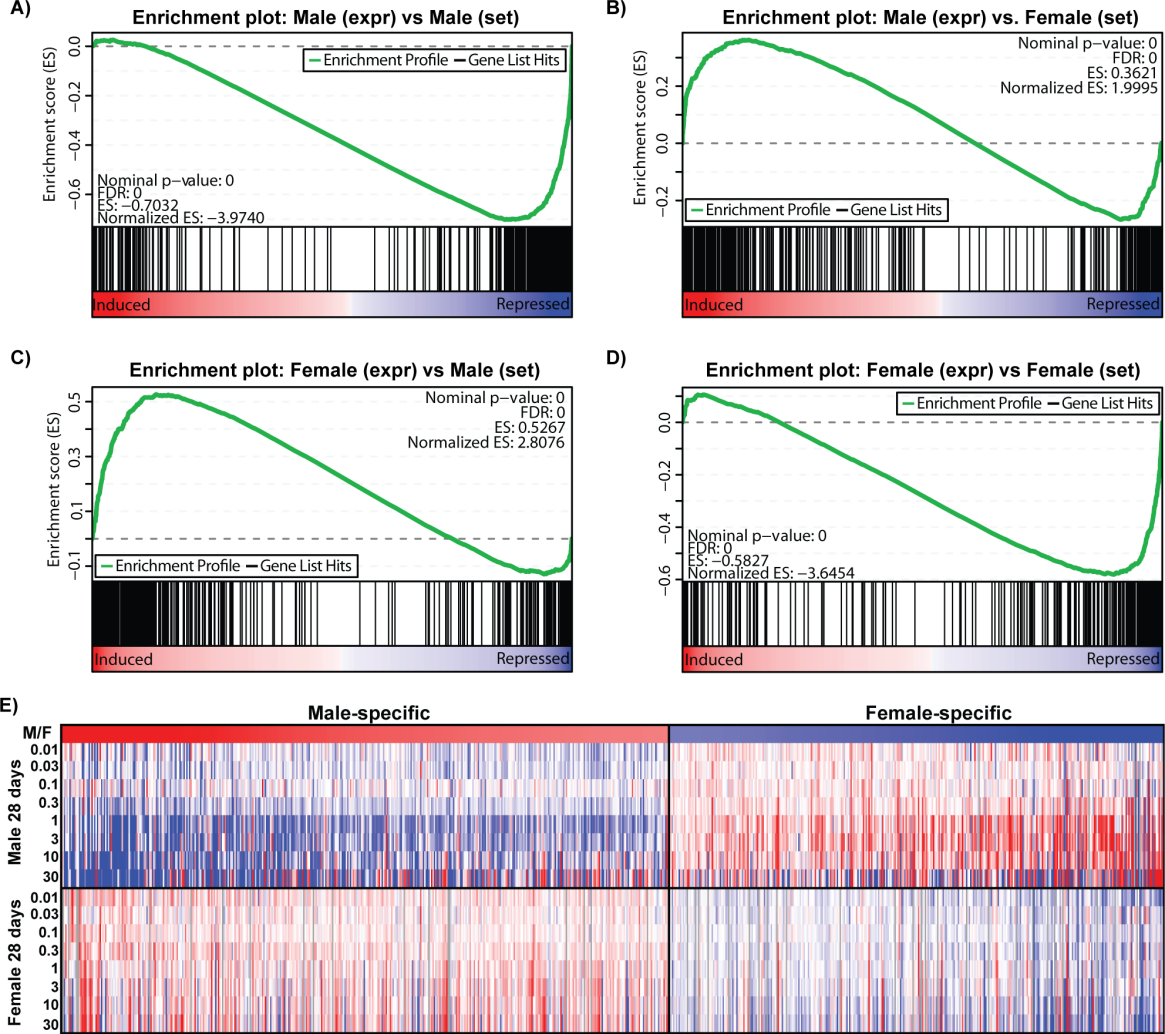
Gene expression changes of sexually dimorphic genes. Gene set enrichment analysis (GSEA) of sexually dimorphic genes in (A-B) male and (B-C) female mice gavaged with TCDD every 4 days for 28 days. GSEA was performed using male-enriched and female-enriched gene sets determined as described in materials and methods. Gene expression changes were ranked from largest positive to largest negative. The top panel (green line) represents a ‘running-sum statistic’ which increases when a gene is in a gene set (denoted by black bars), and decreasing when it is not. TCDD repressed the majority of male-specific dimorphic genes (A) while inducing many female-specific genes (B) in male mice. Conversely, TCDD induced many male-specific genes (C) while repressing a majority of the female-specific genes (D) in female mice. (E) Heat map demonstrates the gene expression changes in male-specific and female-specific genes at 28 days.

**Fig 8:**
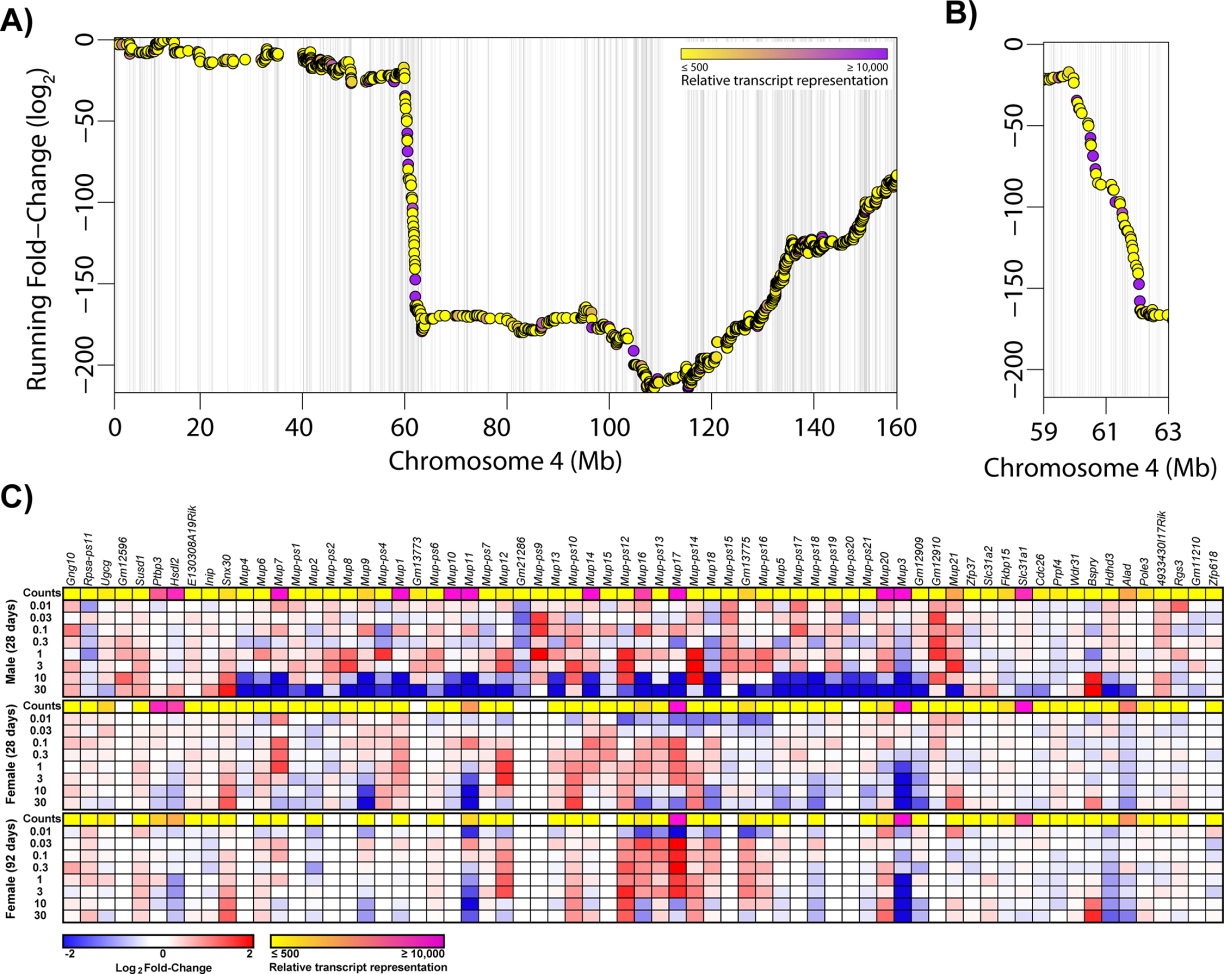
Coordinated repression of *Mup* genes on chromosome 4. (A) Running fold-change for chromosome 4 was calculated for male mice gavaged with TCDD every 4 days for 28 days. Points are colored based on maximum read counts with yellow representing lower expression (≤ 500 reads) and pink representing higher expression (≥ 10,000). The presence of AhR ChIP-Seq peaks at 2h in males is indicated by light grey vertical bars behind points. (B) A region demonstrating a dramatic decrease in the Running fold-change was magnified identifying a chromosomal region with multiple genes repressed by TCDD. (C) Genes in the magnified region are show as heat maps.

Liver-specific gene expression is also regulated by sexually dimorphic transcription factors [17, 19, 20, 26]. Accordingly, many liver-specific genes are also sexually dimorphic [14]. Hepatic transcription factors implicated in sexual dimorphism such as *Onecut1*, *Stat5a*, *Stat5b*, and *Bcl6* were repressed 5.6-, 2.5-, 2.0, and 4.8-fold, respectively, by TCDD in males (Fig 6A and 6B). *Cux2*, a female-specific transcription factor exhibited dose-dependent repression (6.2-fold) in female mice at 92d. Analysis of male and female RNA-Seq data identified 442 male-enriched genes and 368 female-enriched genes using a |fold-change| ≥ 2.0 and P1(*t*) ≥ 0.8 criteria. Sulfotransferases (e.g. *Sult2a1*, *2a2*, *2a5*, *3a1*) exhibit female-specific expression while *Cyp4a12* and several mouse urinary proteins (e.g. *Mup7*, *11*, *20*, and *21*) are primarily expressed in males [40, 41]. A total of 26 genes were classified as sexually dimorphic and liver-specific (S3 Fig). GSEA revealed TCDD repressed male-specific genes while female-specific genes were induced in male mice, and vice-versa in females (Fig 7). For example, TCDD divergently regulated *Gstp1* (induced up to 4.5-fold in females; repressed down to 3.0-fold in males) and *Cyp2d9* (induced up to 9.8-fold in females; repressed down to 5.8-fold in males). GSTP1 dimorphic expression was confirmed at the protein level (Fig 2). In contrast, highly expressed (>10,000 read counts) major urinary protein genes were repressed in both male (*Mup1*, *3*, *10*, *11*, *14*, *17* and *20* were repressed 167-, 1,538-, 100-, 270-, 179- and 90.9-fold, respectively, at 30 μg/kg) and female (Mup*3*, *11* and *17* repressed 25.0-, 9.1 and 2.2-fold, respectively, at 30 μg/kg) livers (Fig 8). Female *Mup1*, *10*, *14*, and *20* are expressed at lower levels (<1700 counts) and were largely unaffected by TCDD. *Mup*s are located on chromosome 4 between 59,956,806 and 62,150,841 where 44 AhR enrichment peaks may be involved in their coordinated repression. The 1.6-fold inhibition of *Zhx2* may also contribute to *Mup* repression [20, 42].

Sexually dimorphic gene expression is mediated, in part, by GH, which is secreted intermittently in males and continuously in females. GH signaling is disrupted by AhR-mediated repression of GHR-JAK2-STAT5a/b signaling [19, 22, 25]. In our study, *Ghr* was repressed 8.3- and 2.4-fold in male and female mice at 30 μg/kg, respectively. Corresponding decreases in STAT5 protein were confirmed (Fig 6C and 6D). Interestingly, HNF4α and STAT5b also regulate a shared set of sexually dimorphic genes [43] including male-specific *Gstp1*, *Cyp2d9*, *Cyp7b1*, *Mup4*, *Cyp4a12*, *Mup1*, *Hsd3b5*, *Elovl3*, and *Slco1a1* which were repressed (3.0- to 1,186-fold) in both sexes, while *Cyp2a4*, *Sult1e1*, and *Cyp2b9* were induced (865.4-, 83.8-, and 28.5-fold, respectively). Further highlighting the convergence of sexually dimorphic adult liver gene expression is the coordinated repression of male-enriched adult hemoglobin subunits *Hba-a1*, *Hba-a2*, *Hbb-bs*, and *Hbb-bt* in males (4.5-, 3.7-, 4.5- and 6.3-fold, respectively), and their induction in females (1.7-, 1.7-, 1.8-, and 2.0-fold, respectively) at 28d. TCDD has been reported to reduce hemoglobin levels in male mice [5].

## Discussion

TCDD induces a broad spectrum of hepatic pathologies from hepatic fat accumulation to HCC in a species-, strain-, sex-, age-, tissue- and cell-specific manner [4–10, 26]. Much remains to be understood about the role of sex in liver disease and toxicity [14]. Consequently, we investigated the effect of TCDD on liver-specific and sexually dimorphic gene expression using hepatic RNA-seq datasets from male and female mice [7, 8, 28]. Overall, gene expression changes elicited by TCDD indicate a loss of liver- and sex-specific gene expression producing a sexually ambiguous and functionally de-differentiation liver.

The dose-dependent repression of liver-specific gene expression suggests diminution of the functional hepatic phenotype. Furthermore, AFP induction suggests the TCDD treated liver is retreating to a less differentiated transcriptome consistent with increases in hepatic and serum AFP levels typically associated with HCC or liver regeneration [44]. AFP induction in differentiated hepatocytes marked by vacuolization suggests TCDD caused a loss in hepatocyte differentiation as opposed to progenitor cell proliferation in response to liver regeneration needs. Moreover, AFP staining in liver cancer is only reported to be cytoplasmic [44] while both intranuclear and cytoplasmic staining was observed with TCDD in males at 30 μg/kg, the significance of which is unclear. Paradoxically, *Alb*, which is typically reciprocally regulated compared to *Afp*, was repressed as expected, yet hepatic protein levels were increased. Interestingly, plasma ALB levels are lower in TCDD exposed waste incineration workers [45] and PCB exposed transformer repair workers [46].

Further evidence of diminished liver function includes the repression of albuminoids, hemoglobin subunits, major urinary proteins, and hepatokines [36, 47]. In addition to participating in the transport of vitamins, lipids, amino acids, steroids and metal ions, albuminoids also bind heme reducing lipoprotein oxidation and oxidative stress [7, 47]. *Alb* repression along with hemoglobin *Hba* and *Hbb* subunits is also consistent with TCDD elicited decreases in serum proteins [5]. Similarly, fetuin-A (*Ashg*) and leukocyte derived chemotaxin 2 (*Lect2*) repression enhances insulin signaling [36] and may support improved glucose tolerance in the absence of altered insulin levels in TCDD treated animals [27]. The physiological significance of *Mup* repression is unclear as hepatic secretion is largely male specific and implicated in lipid and pheromone binding. Although *Mup*s also regulate glucose and lipid metabolism by repressing gluconeogenic and lipogenic genes [48], they are not expressed in humans.

There was also concomitant loss of sexually dimorphic gene expression consistent with reports of male liver “feminization” and divergent regulation of sexually dimorphic genes [12, 13, 26]. Our comparative study indicates loss of sexually dimorphic expression as opposed to feminization or masculinization. This is likely due to the combined loss of both male gene repression and female gene induction in females, and vice-versa in males. Notably, the number of differentially expressed genes in TCDD treated males (9,314) was much larger than in females (3,766) [7, 12, 13]. This large difference in the number of dysregulated genes between sexes was also reported for HNF4α null mice which resulted in ~1,000 fewer DEGs in female mice [43]. HNF4α regulates liver-specific and GHR-JAK2-STAT5-mediated sexually dimorphic gene expression, the latter of which is impaired by AhR activation through repression of *Ghr* and *Jak2* [17, 19, 22, 26]. Moreover, mouse models lacking hepatic GHR, JAK2, or STAT5 have increased liver damage and develop features of NAFLD [49] suggesting GHR-JAK2-STAT5 impairment contributes to TCDD-elicited NAFLD. The anti-estrogenic effects of AhR agonists in mediating these changes remains unclear [50, 51].

The repression of the negative regulator ZHX2 may also be involved in the loss of liver-specific and sexually dimorphic gene expression [20, 52, 53]. ZHX2 inhibits fetal gene expression in adult liver, namely *Afp*, *H19*, and *Gpc3*, as well as several sex-specific cytochrome P450s [20, 21, 53, 54], all of which were induced by TCDD. Similarly, ZHX2 is required for high expression of *Mups* [42] most of which were repressed by TCDD. Overall, ZHX2 repression is consistent with the loss of tissue- and sex-specific gene expression and the induction of fetal genes commonly associated with HCC.

Collectively, our studies suggest that TCDD elicits a loss of liver identity by diminishing both tissue-specific and sexually dimorphic gene expression. While the exact mechanism remains to be elucidated, impairment of GHR-JAK2-STAT5 signaling, dysregulation of HNF-mediated transcription, and *Zhx2* repression are implicated. These effects may also contribute to the development of NAFLD pathologies and other AhR-mediated toxicities. Indeed, the repression of HNF6 (*Onecut1*) which plays a regulatory role in sexually dimorphic and liver-specific gene expression, also regulates hepatic lipid metabolism via induction of the transcriptional repressor REV-ERBα (*Nr1d1*) [55] which was induced by TCDD [7]. In summary, persistent AhR activation promoted the loss of unique hepatic functions and sexual dimorphism that may factor in TCDD-elicited NAFLD and HCC.

## Supporting information

S1 Table Gene expression changes of sexually dimorphic genes. (XLSX)

S2 Table Gene expression changes in TCDD treated mice for 181 liver-specific genes. (XLSX)

S1 Fig Generation of liver-specific gene set. Microarray datasets for basal gene expression for 96 different male mouse tissues/cell types were obtained from Lattin *et al*. [34]. The difference between the microarray signal in the liver and the maximum fluorescent signal on a per gene basis in all other tissues/cells was calculated (ΔSignal). A gene was considered liver-specific when ΔSignal ≥ 5,000 units.
(DOCX)

S2 Fig Chemiluminescence traces of Wes protein assays. Data was collected and analyzed as described in materials & methods.
(DOCX)

S3 Fig Comparison of liver-specific and sexually dimorphic gene sets. (DOCX)
