# Supplementary figures and images for: Loss of liver-specific and sexually dimorphic gene expression by aryl hydrocarbon receptor activation in C57BL/6 mice
Source: PLoS One. 2017 Sep 18;12(9):e0184842. doi: 10.1371/journal.pone.0184842 (PMC5602546; doi:10.1371/journal.pone.0184842)

**FIGURE S1**

**
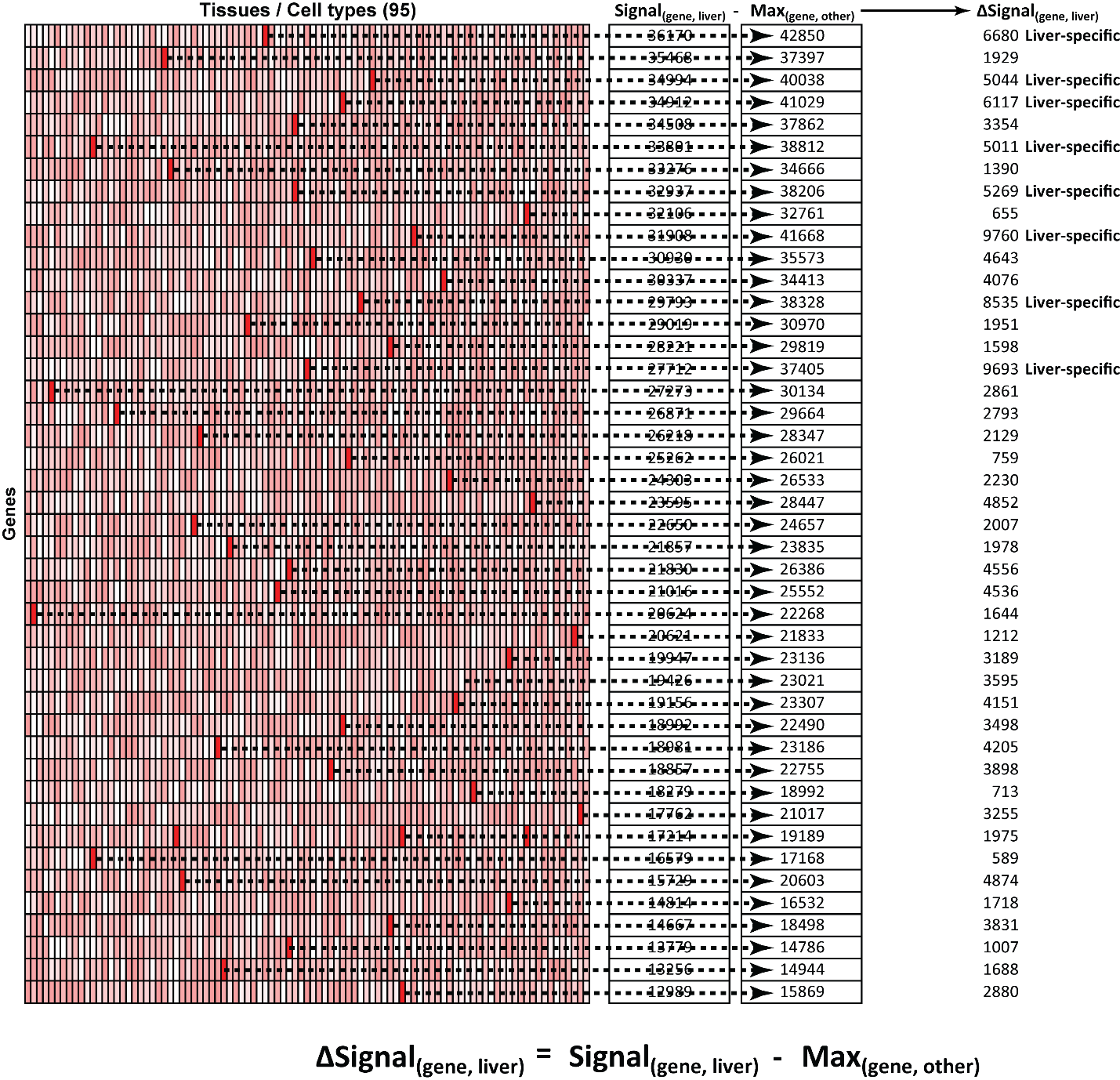
**

Supplement: S1 Fig — Microarray datasets for basal gene expression for 96 different male mouse tissues/cell types were obtained from Lattin et al. [34]. The difference between the microarray signal in the liver and the maximum fluorescent signal on a per gene basis in all other tissues/cells was calculated (ΔSignal). A gene was considered liver-specific when ΔSignal ≥ 5,000 units. (DOCX) [file pone.0184842.s003.docx]

**FIGURE S2**

**
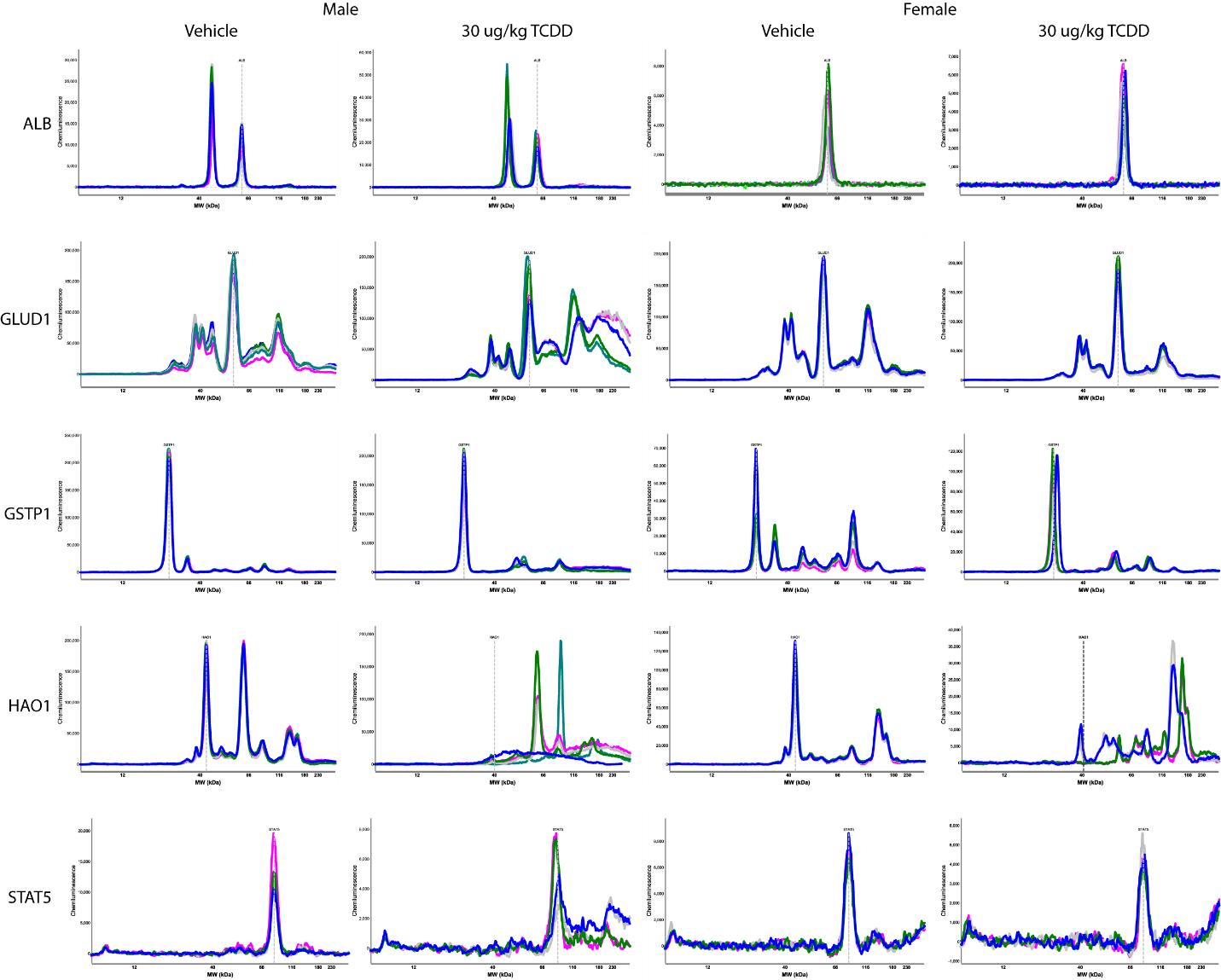
**

Supplement: S2 Fig — Data was collected and analyzed as described in materials & methods. (DOCX) [file pone.0184842.s004.docx]

**FIGURE S3**

**
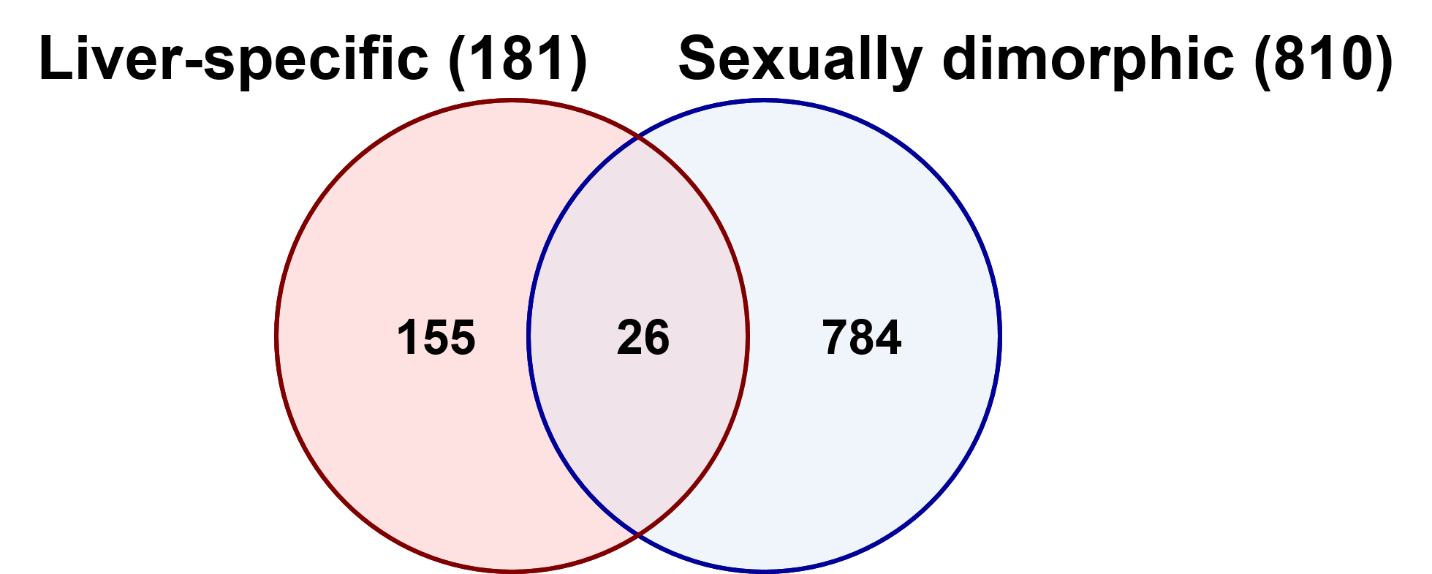
**

Supplement: S3 Fig — (DOCX) [file pone.0184842.s005.docx]
